# The effect of Jordanian essential oil from coriander seeds on antioxidant, anti-inflammatory, and immunostimulatory activities using RAW 246.7 murine macrophages

**DOI:** 10.1371/journal.pone.0297250

**Published:** 2024-08-06

**Authors:** Amin Omar, Muna Barakat, Lujain F. Alzaghari, Shaymaa B. Abdulrazzaq, Eliza Hasen, Dinesh Kumar Chellappan, Mohammad A. A. Al-Najjar

**Affiliations:** 1 Faculty of Pharmacy, Department of Pharmaceutics and Pharmaceutical Sciences, Applied Science Private University, Amman, Jordan; 2 Faculty of Pharmacy, Department of Clinical Pharmacy and Therapeutics, Applied Science Private University, Amman, Jordan; 3 Department of Chemical and Pharmaceutical Sciences and Biotechnology, Chemical Sciences Division, Chemistry Interdisciplinary Project (ChIP), University of Camerino, Camerino (Macerata), Italy; 4 Department of Life Sciences, School of Pharmacy, International Medical University, Bukit Jalil, Kuala Lumpur, Malaysia; Sam Higginbottom Institute of Agriculture, Technology and Sciences, INDIA

## Abstract

Coriander (*Coriandrum sativum L*.) is a member of the Umbelliferae/Apiaceae family and one of the well-known essential oil-containing plants, in which the seeds are used in traditional medicine, and as flavoring in food preparation. Knowing the diverse chemical components of different parts of the plant, this work aims to investigate the antioxidant, the anti-inflammatory, and the immunostimulatory modulator effects of the Jordanian *C*. *sativum*’s seed extracted essential oil (JCEO). Coriander oil extract was prepared by hydro-distillation method using the Clevenger apparatus. Different concentrations of coriander oil were examined by using DPPH radical scavenging assay, MTT assay, pro-inflammatory cytokine (Tumor Necrosis Factor-TNF-alpha) production in RAW264.7 murine macrophages in addition, scratch-wound assessment, NO level examination, Th1/Th2 assay, phagocytosis assay, and fluorescence imaging using DAPI stain were conducted. JCEO had a potential metabolic enhancer effect at a concentration of 0.3 mg/mL on cell viability with anti-inflammatory activities via increasing cytokines like IL-10, IL-4, and limiting NO, INF-*γ*, and TNF-*α* release into cell supernatant. Antioxidant activity was seen significantly at higher concentrations of JCEO reaching 98.7% when using 100mg/mL and minimally reaching 50% at 12.5mg/mL of the essential oil. Treated macrophages were able to attain full scratch closure after 48-hrs at concentrations below 0.3mg/mL. The seed-extracted JCEO showed significant free radical scavenging activity even at lower dilutions. It also significantly induced an anti-inflammatory effect via an increase in the release of cytokines but reduced the LPS-induced NO and TNF-α production at 0.16–0.3mg/mL. In summary, coriander essential oil demonstrated antioxidant, anti-inflammatory, and immunostimulatory effects, showcasing its therapeutic potential at specific concentrations. The findings underscore its safety and metabolic enhancement properties, emphasizing its promising role in promoting cellular health.

## Introduction

Medicinal plants have been utilized for thousands of years to cure many different diseases. They are currently used by a large percentage of the population because of their generally reasonable safe application, considerable customer acceptability, and possible multi-purpose functional use [1]. Nowadays, we can state that bioactive components, extracted from plants along with their chemical substitutes, account for more than half of FDA-approved medications and continue to be significant sources for the discovery of novel active pharmaceuticals [2].

Plants that produce essential oils represent a great portion of the abundant natural resource used in a variety of industries, including pharmaceutical, food, and cosmetic fields. This is due to their flavor, smell, and diverse biological activity [3]. One of these remarkable plants is coriander. Coriander (*Coriandrum sativum L*.) is classified as a member of the Umbelliferae/Apiaceae family that is widely utilized in pharmaceuticals and food industries as a herb and medicine [4]. It is well established that coriander possesses high concentrations of bioactive substances such as linalool and geranyl acetate, which are known for their antioxidant, anti-inflammatory, and antibacterial properties [5–8]. Furthermore, previous research conducted on coriander revealed potential anti-diabetic, hypocholesterolemic, anthelmintic, hepatoprotective, antihypertensive, anticancer [9,10], and interestingly, some studies showed a protective effect against metals poisoning [11,12].

Inflammation is a "host defense" strategy against pathogens that involves increased or excessive production of Reactive Oxygen Species (ROS) by activated pro-inflammatory cytokines and immune cells [13]. ROS aids in clearing tissue invading germs, but when produced in large quantities, it can increase oxidative stress and chronic inflammatory-related diseases [14]. Furthermore, while inflammation causes oxidative stress, the inverse sequence of events is also true. As a result, inflammation and oxidative stress are tightly intercalated [15].

Severe inflammation can increase immune cell activity, which may subsequently, damage tissues and body health [16,17]. When inflammation develops, many pro-inflammatory mediators are overproduced, leading to the escalation of a variety of reactions such as vascular changes and white blood cell responses [18]. Macrophage cells have a vital role in the activation of pro-inflammatory mediators. Toll-like receptor 4 becomes active in cases of infected cells, causing nuclear factor-kappa B (NF-*κ*B) translocation from the cytoplasm into the nucleus [19]. Once NF-*κ*B is active, inflammatory mediators such as interleukin-6 (IL-6), tumor necrosis factor-α (TNF-α), Nitric Oxide (NO), and cyclooxygenase-2 (COX-2) are activated [20–23]. However, a recent study showed that coriander oil extract derivatives such as phenolic compounds and flavonoids possess anti-inflammatory action through decreasing the levels of multiple inflammatory cytokines or inflammatory mediators such as IL-1β, IL-6, IL-10, TNF-α, NF-*κ*B, NO, and COX-2 [24].

This study aims to assess the antioxidant, anti-inflammatory, and immunostimulatory activities of different concentrations of Jordanian seed extracted coriander essential oil (JCEO) against RAW246.7 murine macrophages using a variety of well-known immunological assays and anti-oxidant assessment techniques.

## Materials and methods

### Extraction of coriander essential oil from Jordanian coriander seeds using hydro-distillation and Clevenger apparatus

**Fig 1:**
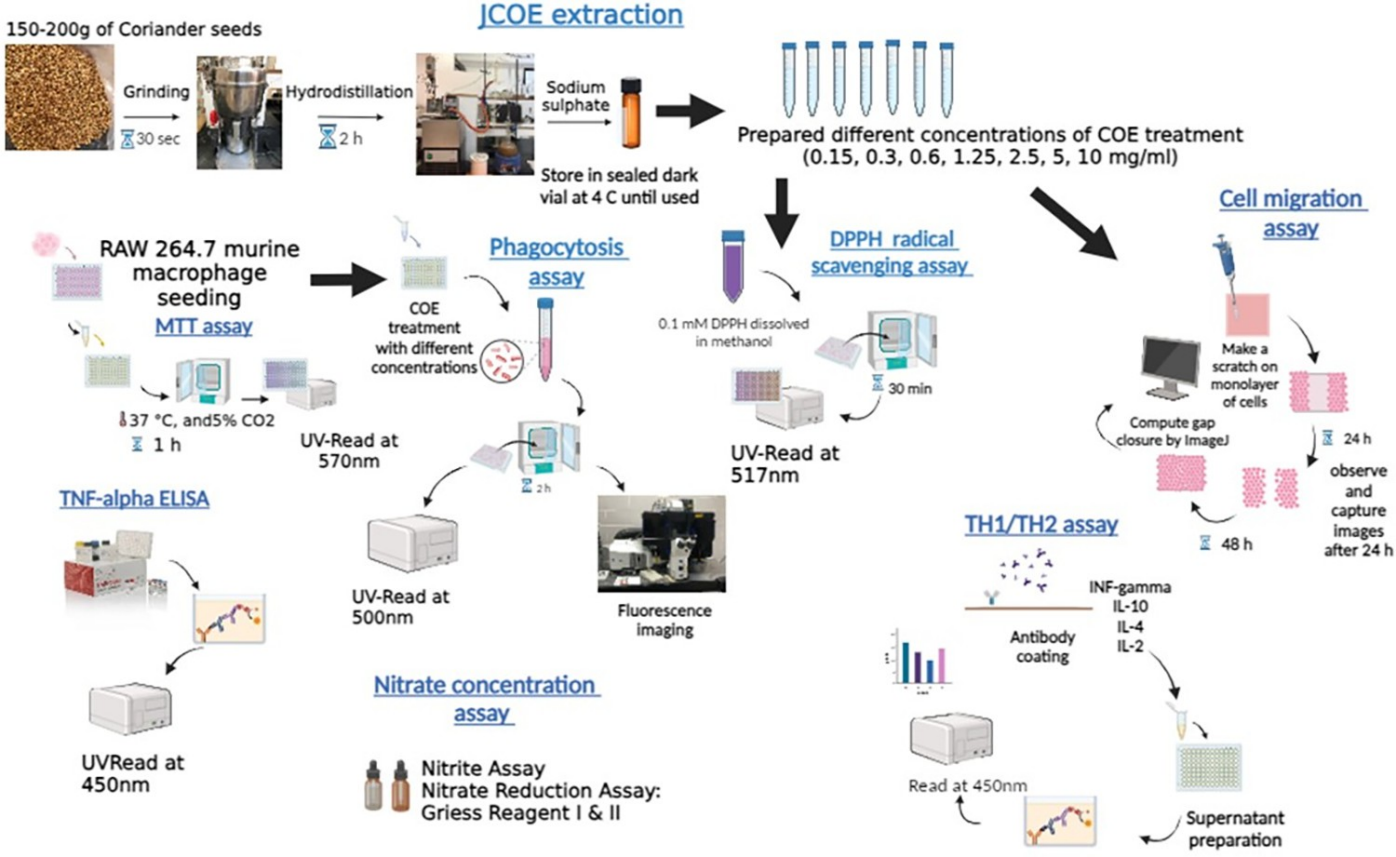
Illustration for the used methods made by using BioRender®.

Coriander seeds were purchased from the Saffron Kingdom herb market in Amman, Jordan. These seeds were cultivated and harvested during 2020–2021. The essential oil from coriander seeds was extracted using a hydro-distillation method and a glass Clevenger apparatus (Borosil, India). The seeds (150-200g) were moderately grinded using the mechanical grinder for 30 seconds before hydro-distillation, to allow more essential oil release. Powdered seeds were mixed with approximately 1500 mL of filtered water and placed in a 2000 mL round-bottom flask to extract the essential oil, followed by heating using a heating mantle (Electrothermal, UK), and condensation using a cooler circulator (Lauda, Austria) for 2-hrs to obtain approximately 1.5-2mL of coriander essential oil [25,26]. The extracted essential oil was collected into dark amber vials and stored at 4±2°C until used for further investigation, Fig 1.

#### Gas chromatography-mass spectrometry (GC-MS) analysis

A sample of the CEO was sent to the University of California, San Diego, USA, for analysis using an Agilent 5977B Gas Chromatograph-Mass Selective Detector (GC-MSD). This instrument features electron impact sources connected to an Agilent 7820A GC system, allowing the analysis of sample mixtures. It is particularly effective for routine examination of non-polar small organic compounds with masses ranging from 50 to 1000 amu. The results were compared against the NIST (National Institute of Standards and Technology) library, which contains over 1 million compounds. For the analysis, approximately 1 μL of the CEO sample was diluted in 10 μL of GC-grade n-hexane and then subjected to GC analysis. This was carried out using a Varian Chrompack CP-3800 GC-MS/MS-200 (Saturn, Netherlands) equipped with a DB-5 (5% diphenyl, 95% dimethyl polysiloxane) GC capillary column (30 m × 0.25 mm i.d., 0.25 μm film thickness) and helium as the carrier gas at a flow rate of 0.9 mL/min. Compounds were identified by comparing them to entries in the built-in libraries (NIST and Wiley Co. USA).

### Cell culturing and treatment

Murine macrophages RAW 246.7 were provided from the American Type Culture Collection (TIB-71™-ATCC, USA). Cells were defrosted from -80±5°C and placed in a 75cm^2^ culture flask in an incubator at 37±2°C and 5% CO_2_. Cells were maintained in Dulbecco’s modified Eagle’s medium (DMEM, Gibco™, USA) supplemented with 10% fetal bovine serum (FBS, Gibco™, USA), and 1% penicillin and streptomycin antibiotic mixture (P/S, Gibco™, USA). The confluence of the cells was continually checked under optical microscopy (Nikon, USA) until it reached 75–80% approximation. Before treatment with JCEO, RAW264.7 cells were seeded at an inoculum of 1×10^5^ cells/well in 96-well cell culture plates and incubated at 37±2°C and 5% CO_2_ in a humidified incubator (Biosan, China) for 24±2 hrs. The medium (DMEM supplemented with 10% FBS) was removed and cultured cells were washed 3 times with phosphate buffer saline PBS (PBS, Gibco, USA). Then, 100μL of different concentrations of JCEO (0.08, 0.16, 0.3, 0.6, 1.25, and 2.5 mg/mL) was added, and the cells were again incubated overnight at 37±2°C and 5% CO_2_. Then, the cells were washed again with PBS, and the supernatant was collected for further analysis. Untreated macrophages were utilized as a negative control, while lipopolysaccharides LPS from *Pseudomonas aeruginosa* (LPS, Sigma Aldrich, L9143) was used as a positive control (1μg/mL).

### Viability assessment

To select a range of non-toxic concentrations, the cytotoxicity potential of different concentrations of JCEO was tested in triplicate using 3-(4, 5-dimethyl-2-thiazyl)-2, 5-diphenyl-2H-tetrazolium bromide (MTT) reduction assay [26,27]. Briefly, 100μL of RAW246.7 murine macrophages were seeded at a density of 1×10^5^ cells/well in a 96-well plate with 100μL of fresh supplemented (FBS 10%) DMEM medium per well. Cells were allowed to adhere for 24±2-hrs under standard conditions in a humidified incubator (37±2°C, 5% CO_2_). The medium was removed after 24±2-hrs of incubation and replaced with 100μL of fresh supplemented (FBS 10%) DMEM medium containing different concentrations (0.08, 0.16, 0.3, 0.6, 1.25, and 2.5 mg/mL) of JCEO. For negative control wells, only 100μL of fresh DMEM medium supplemented with FBS 10% and Penicillin/Streptomycin1% (Gibco, UK) was utilized. For positive control wells, only 100μL of supplemented (FBS 10%) DMEM medium with LPS (1μg/mL) was utilized. Following 24±2-hrs of incubation, 20μL of MTT (5 mg/ml; Sigma Aldrich) was added into each well and further incubated for another 4-hrs in a humidified incubator (37±2°C, 5% CO_2_). The formation of insoluble purple formazan from yellowish MTT reaction was dissolved by the addition of 100μL of Dimethyl sulfoxide (DMSO, Sigma Aldrich), and the absorbance was measured at 570nm using a Bio-Tek® microplate reader (Bio-Tek, Germany). The formula below was used to calculate the percentage of cell inhibition:

PercentageofCellViability(%)=(opticaldensityoftreatedcell/opticaldensityofcontrolcell)*100%

### Cell migration assay

RAW246.7 cells with a density of 1×10^5^ cells/well) were seeded in a 96-well plate and grown for 24±2-hrs under standard conditions in a humidified incubator (37±2°C, 5% CO_2_). The cell monolayer was then scratched vertically with a sterile 10μL pipette tip, washed three times with PBS (Gibco, USA), and incubated with 100μL of supplemented (10% FBS) DMEM medium containing JCEO with different concentrations (0.3, and 0.16 mg/mL). LPS (1μg/mL) was utilized as a positive control, while untreated macrophages were used as a negative control. The plate was then incubated at 37±2°C in a humidified 5% CO2 incubator and the gap closure was observed using an inverted lens microscope (Nikon, USA) after 24±2 hrs, and 48±2-hrs. The gap closure was calculated using the ImageJ software using images taken by iPhone (Apple, USA).

### TNF-α assay

Tumor necrosis factor-α (TNF-α) level was determined using a mouse enzyme-linked immunosorbent assay (ELISA) kit (Thermo Fisher, US) according to the manufacturer`s instructions. Treatment of RAW264.7 cells (1×10^5^ cells/well) with different concentrations of JCEO (0.3 and 0.15 mg/mL), side by side with LPS (1μg/mL) as a positive control, while untreated macrophages were used as a negative control. The supernatant was obtained from wells after 6 hr of treatment and the amount of TNF-α in cell-free supernatant was determined at 450 nm.

### The DPPH free radical-scavenging assay

In this test, the 2,2-diphenyl-1-picrylhydrazyl (DPPH) radical, which generates a purple solution (λ max = 517 nm), is reduced when it combines with any antioxidant that can contribute a hydrogen atom, generating a yellow-colored diphenyl-picrylhydrazine molecule [28,29]. The DPPH free radical-scavenging activity assessment was carried out using a previously published method [30,31]. JCEO was diluted in methanol to make concentrations range of 100, 50, 25, 12.5, 6.25, 3.13, 1.6, and 0.78 mg/mL. The 180μL reaction mixture contained 90μL of 0.1mM DPPH (dissolved in methanol) and 90μL of JCEO solutions of various concentrations. The test reagent was mixed with the different JCEO concentrations thoroughly in 96-well plates, incubated at 37±2°C for 30 minutes, and the absorbance at 517 nm was measured using a Bio-Tek® microplate reader (Bio-Tek, Germany). The percentage of DPPH antioxidant activity was calculated as follows:

DPPHScavengingActivity(%)=[(Absoftheblank–Absofthesample)/(Absoftheblank)]*100%

### Cytokine release assessment

Supernatants (100μL) were collected from previously seeded cells at 1×10^5^ cells/well density to assess the levels of INF-γ, IL-10, IL-4, and IL-2 using TH1/TH2 ELISA kit (Thermo Fisher, USA), as per the manufacturer’s protocol. The absorbance readings for each treatment were translated into concentration (ng/mL) using a standard curve of predetermined cytokine concentrations.

### Phagocytosis assay

A phagocytosis assay kit (Red *E*. *coli*, ab235901, UK) was used to measure phagocytic activity. Following 24±2-hrs previously seeded RAW267.4 cells at a density of 1×10^5^ cells/well, followed by treatment of 0.6, 0.3, and 0.16 mg/mL of JCEO, seeded cells were rinsed three times with PBS. A volume of 5μL of red fluorescence *E*. *coli* was placed in each well, and incubated at 37±2°C, 5% CO2 for 2-hrs. Wells were rinsed again three times with 100μL PBS, and fluorescence was measured using a Bio-Tek® microplate reader (Bio-Tek, Germany) at 509⁄533nm.

### Fluorescence imaging using DAPI stain

RAW246.7 murine cells were seeded overnight on coverslips inside a 6-well plate (1×10^5^ cells/well). The next day the cells were treated with different concentrations of JCEO (0.6, 0.3, and 0.16 mg/mL) for 24±2-hrs at 37±2°C incubator. After 24-hrs of incubation, the cells were washed three times using 100μL PBS to remove all DMEM residual before the addition of *E*. *coli* particles (5μL of *E*. *coli* in 100μL of media in each well) for 2-hrs. After applying the *E*. *coli* for 2-hrs, the media was removed and the cells were rinsed with 100μL PBS three times, before they were fixed using 4% formaldehyde prepared in PBS. For staining and imaging purposes, the 4% formaldehyde was discarded, and the cells were washed again with 100μL PBS three times. Two drops of DAPI (4’,6-diamidine-20’-phenyl indole dihydrochloride) solution (abcam, UK) was added to the slide, and the coverslip was removed from the 6-well plate using forceps to place it above the DAPI solution for fluorescence imaging using confocal laser scanning microscope (Confocal LSM 780, ZEISS, Germany).

### Nitrate concentration assay

RAW264.7 macrophages (1×10^5^ cells/well) were previously seeded in 96-wells and pre-treated with LPS (1μg/mL) for 24±2-hrs, and then treated with different concentrations of JCEO (0.6, 0.3, and 0.16 mg/mL) for another 24±2-hrs. The collected supernatant was used to measure the nitrite levels by Griess reagent using a Total NO/Nitrate/Nitrite Parameter Assay kit (Parameter^TM^, R&D Systems, USA) as per the manufacturer’s protocol. The absorbance was detected using a Bio-Tek® microplate reader (Bio-Tek, Germany) at 550 nm.

### Statistical analysis

Data analyses were accomplished by using the GraphPad Prism 9 (GraphPad Software, Inc., San Diego, CA, United States). Data is presented as mean ± SD. Statistical significance was determined using one-way analysis of variance (ANOVA) with Tukey’s post-hoc test for multiple group comparisons. All statistical analyses were based on a *p*-value < 0.05 level of significance.

## Results

### Essential oil characterization

The amount of the CEO obtained from each 100 g coriander seeds was 0.313 (±0.01) mL, (n = 3). GCMS analysis (S1 File) of the hydro-distilled oil from the Coriander seeds revealed 6 identified major volatile compounds: α-pinene (terpene), limonene (aliphatic hydrocarbon), linalool (terpene alcohol), camphor (terpene ketone), o-cymene and γ-terpinene (hydrocarbons), as demonstrated in Table 1.

**Table 1:** Estimations of identified major volatile components from the GCMS peaks of the Coriander essential oil.

Peak	Compound	RT (min)	%
Peak A identification	α-pinene	5.3	2.5%
Peak B identification	o-Cymene	6.6	4.075%
Peak C identification	Limonene	6.8	0.625%
Peak D identification	γ-Terpinene	7.1	1.875%
Peak E identification	Linalool	7.55	86.875%
Peak F identification	Camphor	8.2	2.825%
Peak G identification	Unidentified	10.48	15.625%

### Viability assessment

**Fig 2:**
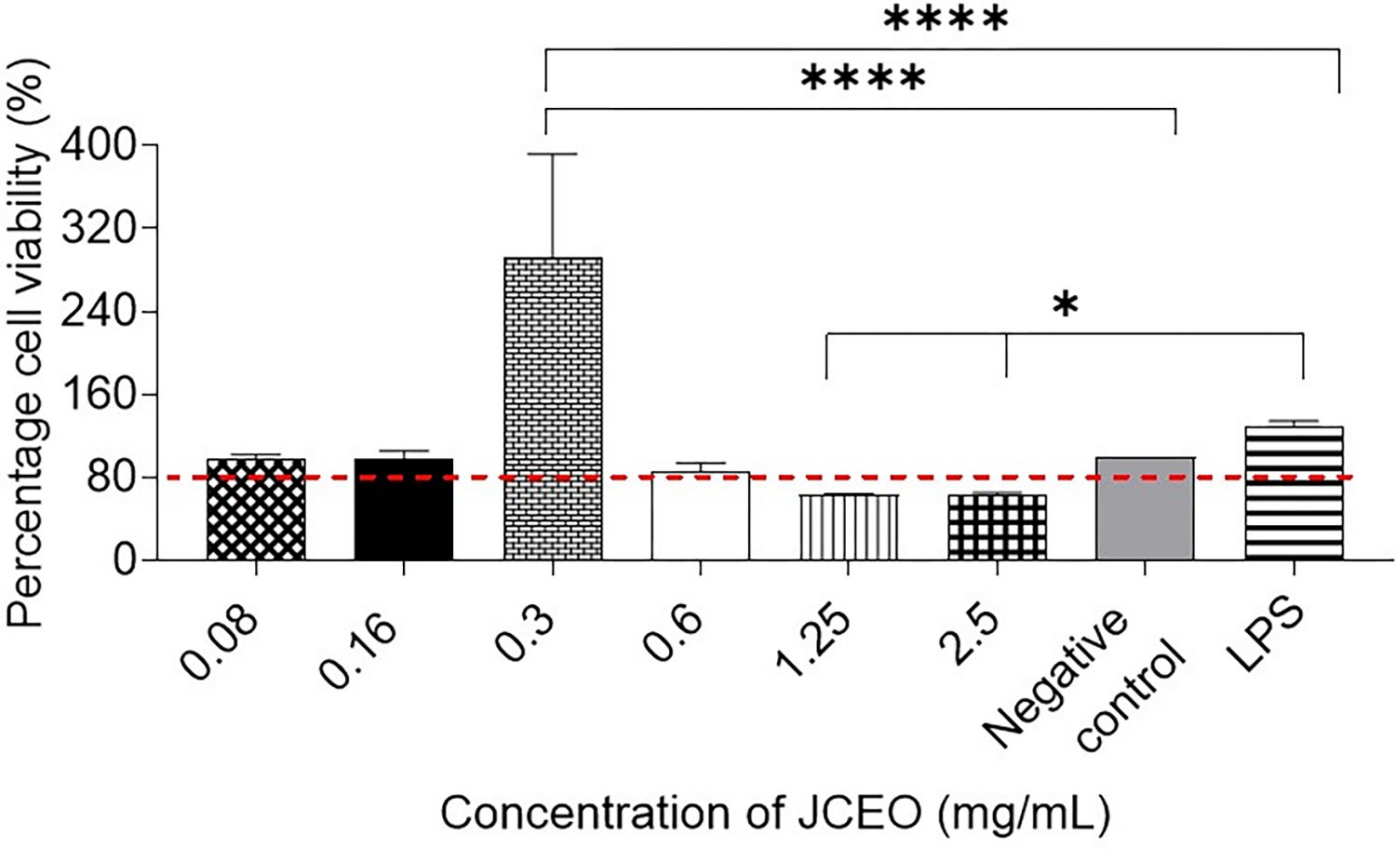
Percentage cell viability of RAW264.7 murine cells 24 hours post-treatment with different JCEO concentrations (0.02, 0.04, 0.08, 0.16, 0.30, 0.60, 1.25 and 2.50mg/mL), positive control LPS and negative control (untreated macrophages). This experiment was conducted using six replicates for each treatment. The lower acceptable limit of cell viability in this study was 80% as indicated by the red dashed line. Data in the figure is represented as mean± SD, (****p <0.0001, * *p*<0.05).

RAW 246.7 murine macrophage cells (1x10^5^ cells/well) were treated with different concentrations of JCEO (0.02, 0.04, 0.08, 0.16, 0.30, 0.60, 1.25 and 2.50mg/mL). The results of the MTT assay showed that concentrations > 0.3 mg/mL of CEO were considerably cytotoxic, as the viability of cells was lower than the accepted viability limit in this study (80%). As demonstrated in Fig 2, a significant increase in the viability percentage of RAW 246.7 was observed at 0.3mg/mL (****p <0.0001). In other words, the percentage had reached 191.85% compared to untreated negative control cells. It was also higher than that of the positive control LPS (1μg/mL), which reached a percentage of 105.38% compared to negative control cells (untreated macrophages).

### Migration assay results

**Fig 3:**
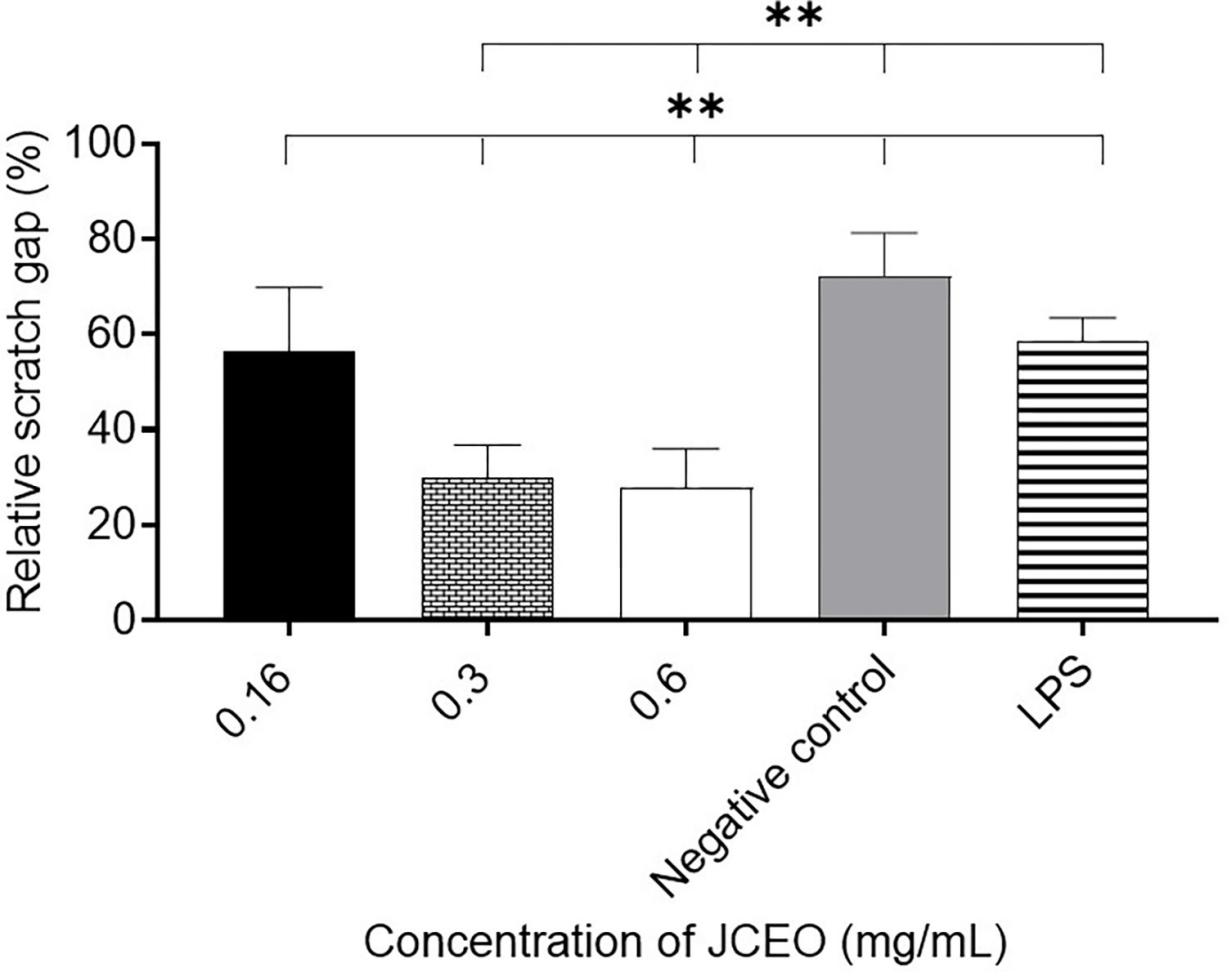
The relative scratch gap percentage of different concentrations of JCEO (0.16, 0.3, and 0.6 mg/mL), positive control LPS (1μg/mL), and negative control after 24 hours. This experiment was conducted using ten replicates for each treatment (**p <0.01). Data in the figure is represented as mean± SD.

RAW264.7 murine macrophages were evaluated in a 96-well pre-seeded plate for 24±2 hrs, and 48±2-hrs after treatment to see the effect of different concentrations of JCEO (0.16, 0.3, 0.6 mg/mL) on cell migration and gap closure. The two concentrations of JCEO 0.3, and 0.6mg/mL treated cells showed the most substantial and maximal gap closure after 24±2 hours when compared to 0.16 mg/mL of JCEO, LPS (1μg/mL), and the negative control (untreated macrophages). However, the concentrations of 0.16, 0.3, and LPS showed a full gap closure after 48 hours, as shown in Fig 3.

### TNF-α assay

**Fig 4:**
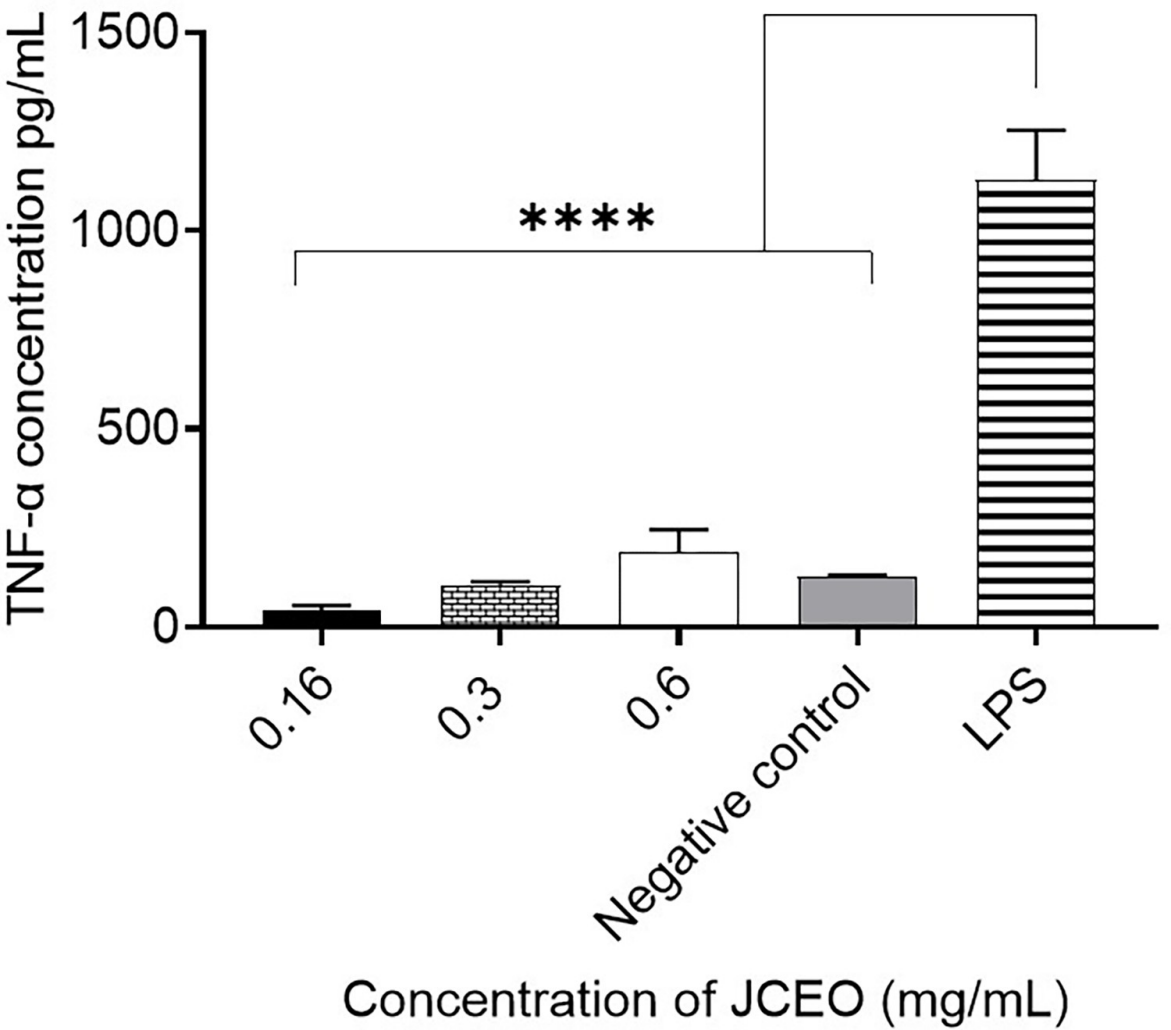
TNF-α concentration in cell supernatant treated with different concentrations of JCOE (0.6, 0.3, 0.16 mg/mL) compared with LPS-treated cells and negative control (untreated macrophages). This experiment was conducted using three replicates for each treatment (*p <0.05, ****p <0.0001). Data in the figure represent mean± SD.

TNF-α was measured in the supernatants of previously seeded RAW264.7 murine macrophages that were treated with different concentrations of JCEO (0.6, 0.3, and 0.16 mg/mL), or LPS (1μg/mL) as a positive control, versus the negative untreated macrophages. The results indicate that there was a significant increase in TNF-α in LPS-activated RAW264.7 cells compared to JCEO (0.6, 0.3, 0.16mg/mL) treated cells and to the negative untreated control (****p <0.0001), as seen in Fig 4. In addition, there was an increase in TNF-α concentration in 0.6 mg/mL JCEO treated cells compared to the untreated macrophages.

### The DPPH radical scavenging assay

**Fig 5:**
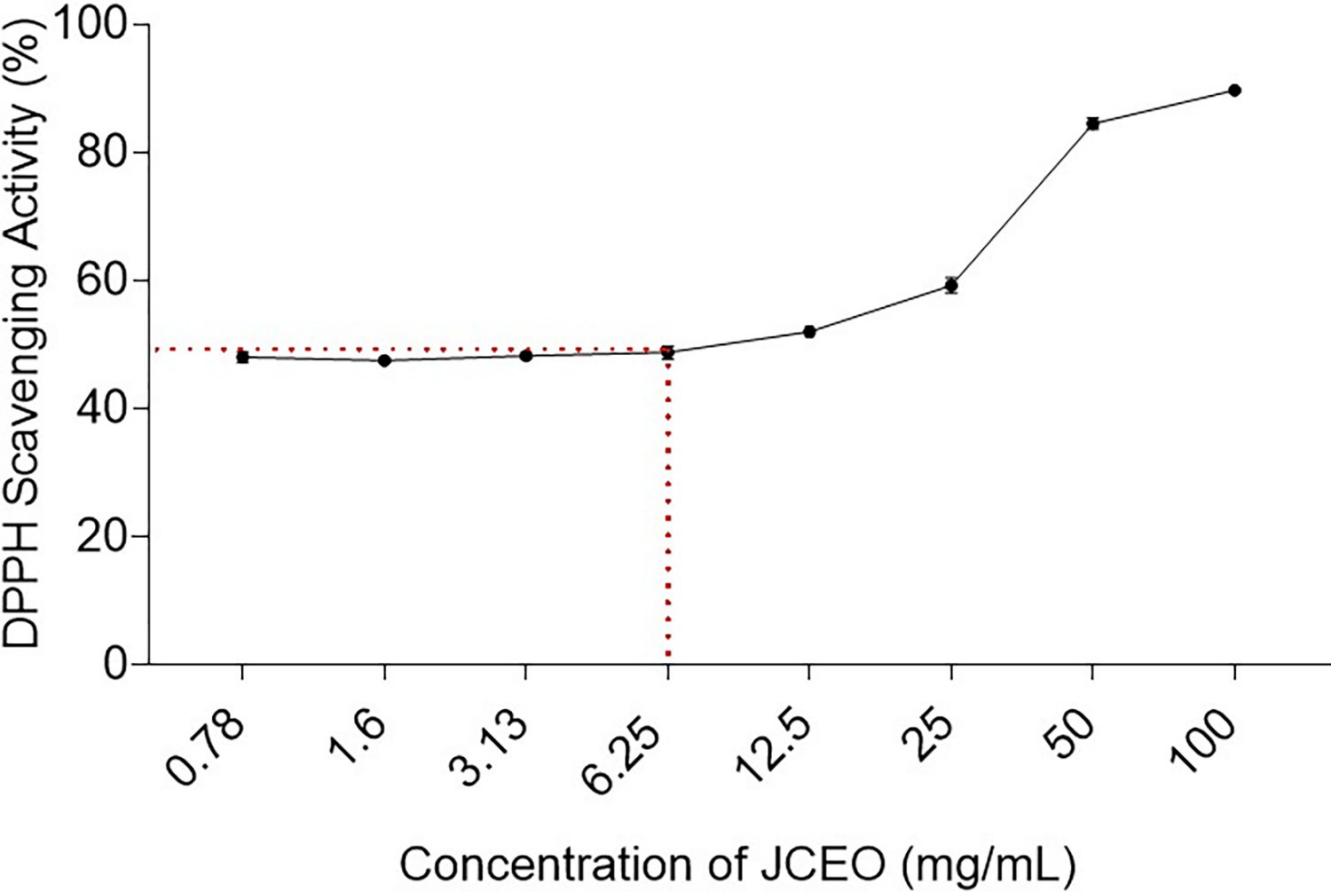
DPPH radical scavenging activity of different concentrations of JCEO (0.78, 1.6, 3.13, 6.25, 12.5, 25, 50, 100 mg/mL) was measured as a percentage value against each concentration of JCEO. Each value was conducted using four replicates for each concentration tested. Data in the figure represented as mean± SD.

DPPH radical scavenging assay was conducted to examine the effect of different concentrations of JCEO (0.78, 1.6, 3.13, 6.25, 12.5, 25, 50, 100 mg/mL) as antioxidants. As can be seen in Fig 5, the highest free radical scavenging activity was shown by JCEO with a concentration of 100mg/mL (98.7%) followed by 50mg/mL (84.4%), 25mg/mL (60.4%), and 12.5 mg/mL (52.2%). It was clear that a reduction in JCEO concentrations resulted in a decrease in antioxidant activity. However, the concentrations of JCEO below 6.26 mg/mL showed no significant change.

### Cytokine release assessment

**Fig 6:**
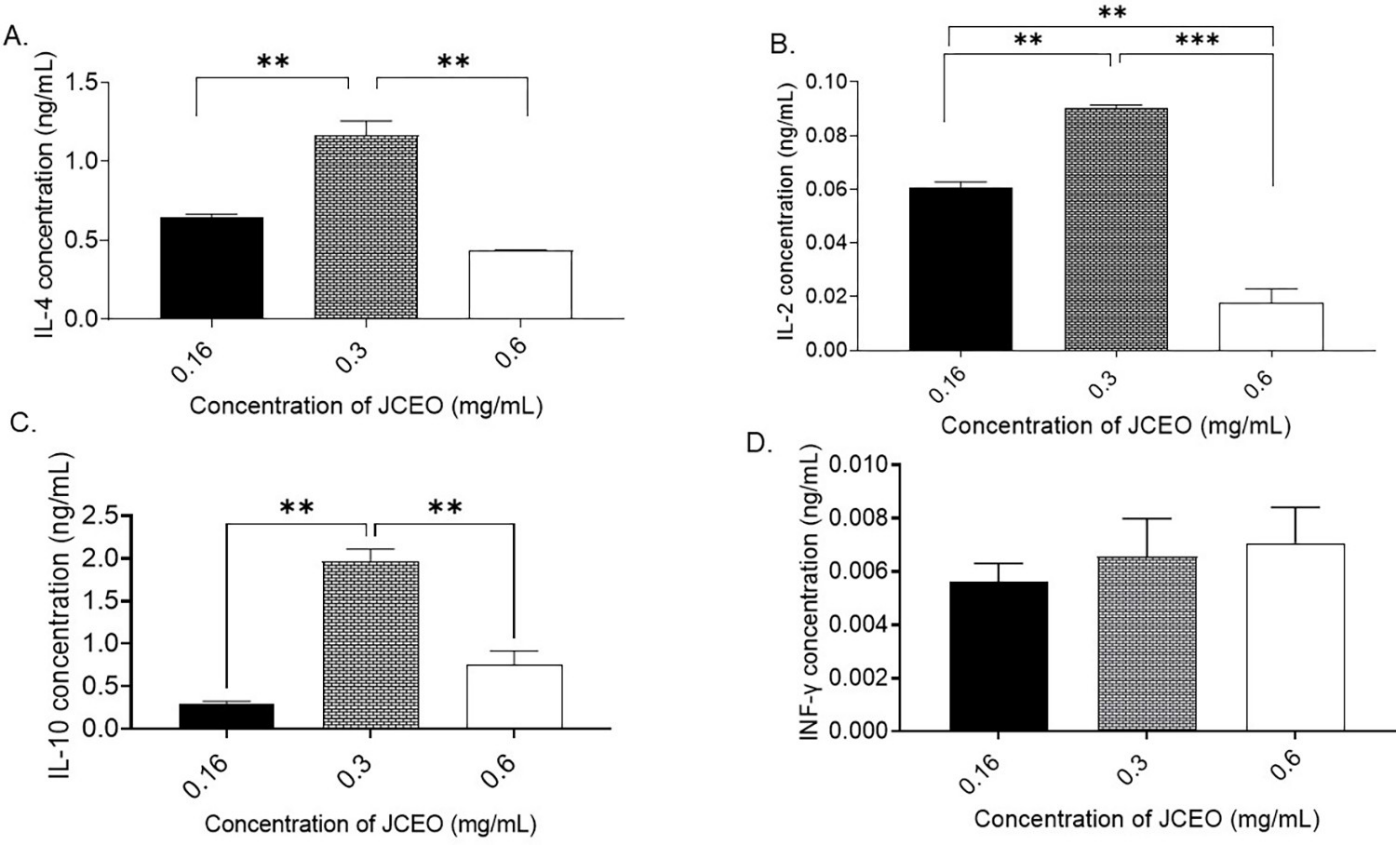
The effect of different concentrations of JCEO (0.6, 0.3, and 0.16 mg/mL) on the concentration level of different cytokines (ng/mL). **A)** INF-γ, **B)** IL-10, **C)** IL-4, **D)** IL-2. This experiment was conducted using a duplicate for each treatment (**p <0.01, ***p <0.001). Data in the figure represented as mean± SD.

The immuno-modulatory effect of different concentrations of JCEO (0.6, 0.3, and 0.16mg/mL) was evaluated by measuring levels of INF-γ (Fig 6A), IL-10 (Fig 6B), IL-4 (Fig 6C), and IL-2 (Fig 6D) using the TH1/TH2 assay kit. The treatment of JCEO induced changes in cytokine levels in the supernatant of RAW264,7 macrophages. Briefly, 0.3 mg/mL JCEO was able to increase IL‐10, IL-4, and IL-2 levels in RAW264.7 murine macrophages compared to 0.6 and 0.16 JCEO treated cells (**p <0.01, ***p <0.001), as shown in Fig 6B–6D, respectively. In addition, 0.16 mg/mL JCEO treated cells increased IL-2 levels in the supernatant of macrophages compared to 0.6mg/mL of JCEO (**p <0.01), as shown in Fig 6D.

### Phagocytosis assay–fluorescence imaging

**Fig 7:**
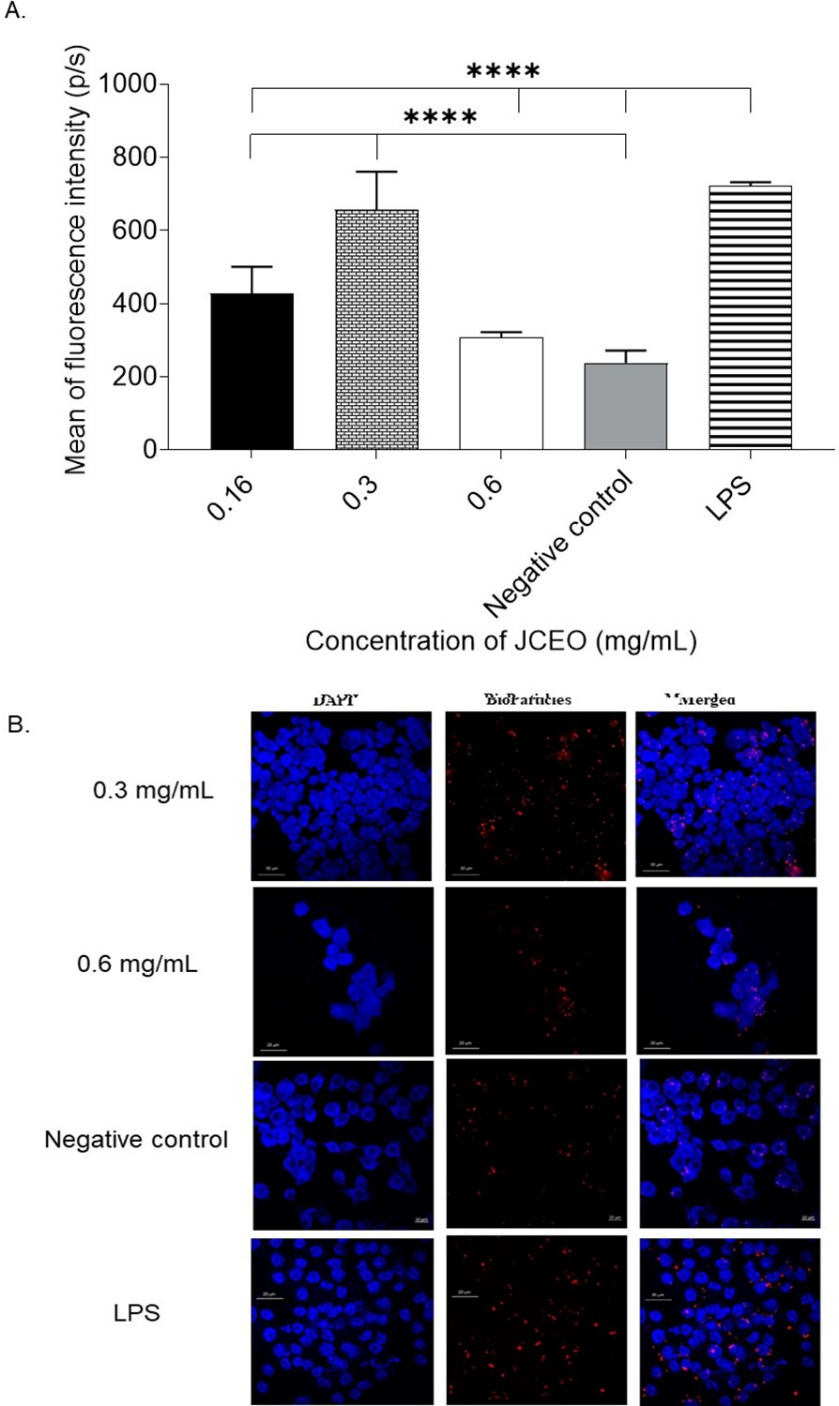
**A)** The mean fluorescence intensity of pHrodo™ Red E. coli BioParticles™ after phagocytosis. This experiment was conducted using a duplicate for each treatment. Data in the figure is represented as mean± SD, ****p <0.0001. **B)** Confocal microscopy images were taken at 10x magnification using confocal LSM 780.

Phagocytosis activity of RAW264.7 cells was measured using fluorescence intensity of phagocytosis after treatment of JCEO with different concentrations (0.16, 0.3, 0.6 mg/mL) when compared to the negative untreated control and the LPS positive control. The 0.3mg/mL concentration of JCEO was seen to have a higher significant mean of fluorescence intensity compared to untreated control and similar intensity to the LPS positive control as shown in Fig 7A (****p <0.0001). Confocal imaging in Fig 7B, confirms the phagocytosis induction in comparison to the control.

### Nitrate concentration assay

**Fig 8:**
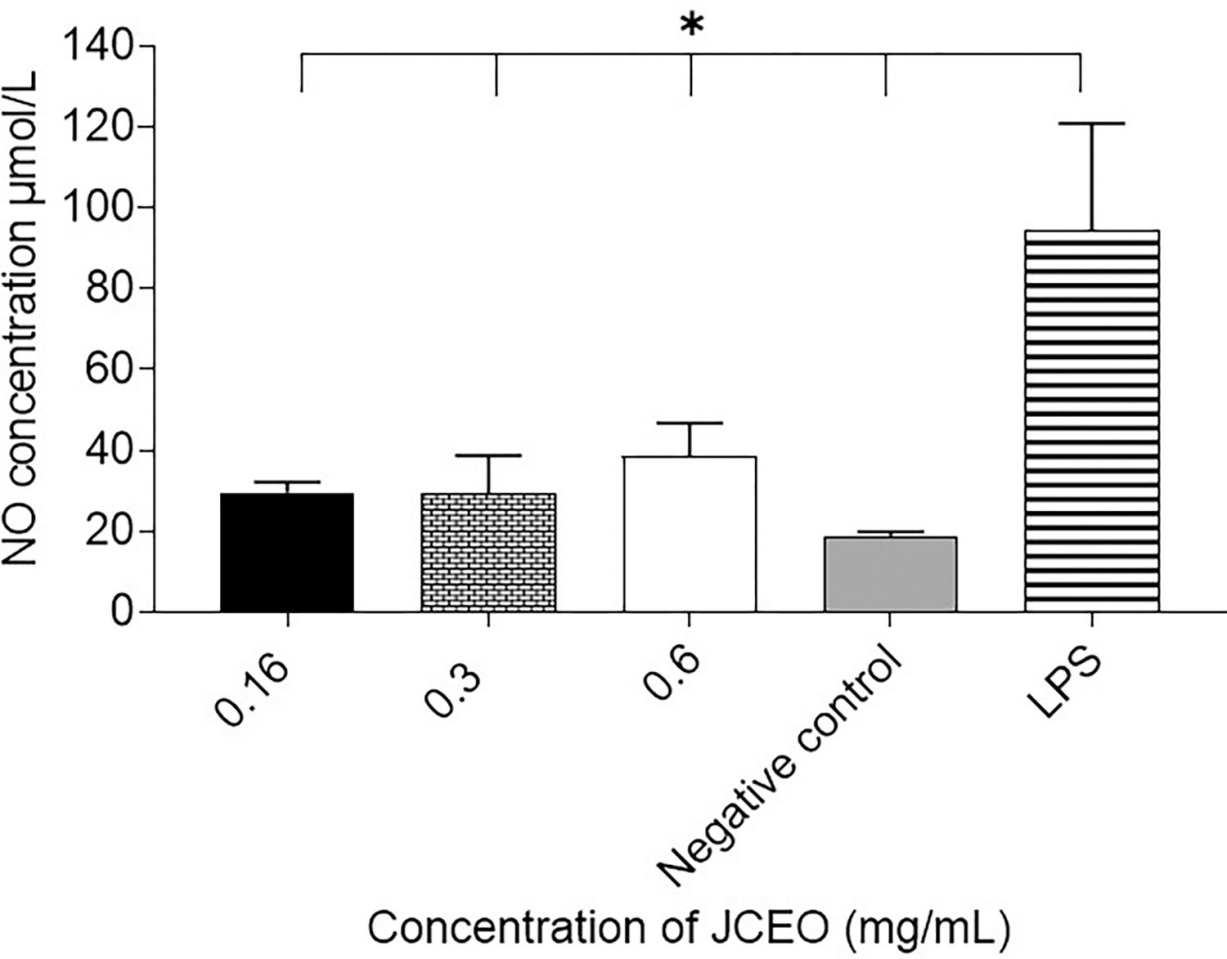
Nitric Oxide concentration in the supernatant of cells collected post 24±2hrs of treatment of LPS-stimulated macrophages with different concentrations of JCEO (0.6, .3, 0.16 mg/mL) compared to LPS 1μg/mL and negative untreated control. This experiment was conducted using a duplicate for each treatment (*p <0.05). Data in the figure are represented as mean± SD.

Different concentrations of JCEO (0.16, 0.4, 0.6 mg/mL) were investigated for the regulation of NO production in LPS-stimulated macrophages, since it is vital to regulate the overproduction of NO, which generates pro-inflammatory responses, in pro-inflammatory conditions. The NO levels were determined using the Griess reagent. RAW264.7 cells were treated with 1 μg/mL of LPS as positive control whereas negative control cells were left untreated. NO production was calculated as nitrite concentration in the supernatant of cells. It was noticed that different concentrations of JCEO (0.6, .3, 0.16 mg/mL) inhibited the production of NO compared to LPS-treated cells (*p <0.05), as demonstrated in Fig 8.

## Discussion

Over the last decade, essential oils have gained attention as a source of bioactive compounds, with many potential health benefits for the essential oil and their bioactive constituents [32–35]. Scholars across the world have attempted to study the chemical composition of essential oils from coriander seeds, using different methodologies [36]. The essential oil content is around 0.18 to 1.40% (v/w) of the coriander seed [37]. The major component that has been previously reported in the oil is linalool, in the range of 60–70% of total seed oil [37,38]. That range is dependent on the region of cultivation, degree of fruit ripening, and time of harvesting [37,39–43]. As seen in our results, Jordanian coriander essential oil (JCEO) extracted from seeds had a high scavenging percentile of free radicals at higher concentrations reaching up to 100mg/mL of oil concentration with 98.7% percentile and decreases with reducing the concentration of JCEO. This shows how our test results are in alignment with previous data on antioxidant activity showing that some essential oils like *Allium cepa L*., *Citrus aurantium*, *Myrtus communis*, *Coriandrum sativum L*., *and Eucalyptus oleosa* revealed a clear antioxidant potential but displayed different ranges of efficacy [40,43]. Essential oils extracted from aerial parts of coriander, specifically the one cultivated in European countries, possessed the highest free radical scavenging capacity using the DPPH scavenging assay [44–48]. The findings of our study suggest that the essential oil from Jordanian coriander seeds in particular could be of interest in the potential prevention of lipid oxidation and binding to reactive oxygen species (ROS) in the human body, preventing oxidation damage or oxidative stress [48,49]. Such natural antioxidants could serve as physical barriers, inhibiting the access of ROS to biological sites and binding to damaging metallic ions [48]. Moreover, linalool is often conveyed as the main factor of the coriander essential oil bioactivity [49], as it was previously proposed that mono- and sesquiterpenes present in the essential oil were responsible for the antioxidant and anti-inflammatory activities [49,50].

JCEO significantly induced an anti-inflammatory effect that was seen in cytokine assay through monitoring the release of IL-10 and IL-4 in the supernatant of treated murine macrophages, where 0.3mg/mL of JCEO had the most noteworthy upstroke of cytokines tested. In addition, LPS-induced macrophages releasing NO were treated with 0.3mg/mL and 0.16mg/mL JCEO having the most limiting concentrations to the release of NO into supernatant. Meanwhile, TNF-α and INF-*γ* have been seen to be low and insignificant levels in our study outcomes. This can be allied to many studies that have confirmed that reactive oxygen species (ROS) are involved in response to inflammation [51,52]. Thus, the enhanced generation of ROS exacerbates inflammation and leads to tissue injury; therefore, preventing the NF-*κ*B signaling pathway, MAPK pathways, TNF-α, INF-*γ*, and ROS generation might be a potential strategy to attenuate inflammatory diseases [51,53]. Our results are in agreement with results from a study about coriander oil components showing high anti-inflammatory activity with IC_50_ of 6.25μM [51,52]. This study demonstrated an inhibitory effect of JCEO on nitrite oxide (NO) level, the LPS-stimulated generations of ROS, and the inflammatory cytokines like IL-6 and TNF-α [51,52]. Moreover, the use of coriander as an anti-inflammatory agent is marked in one of the traditional formulations from Sri Lanka, containing coriander seeds as one of its primary components. Administration of this formula inhibited carrageenan-induced rat paw edema and augmented pain tolerance in rats by 57% after 1-hr treatment as evaluated by the hot plate test [35,54–57]. This formula was examined on patients suffering from rheumatoid arthritis for 3 months and it was noted that there was an improvement in pain, inflammation, and mobility without any adverse effects on liver functions and gastrointestinal functions [57]. Another study on a multi-herbal formulation, consisting of coriander as one of the constituents, confirmed the inhibitory effect against inflammatory bowel disease [58]. The topical anti-inflammatory effect of coriander oil was also reported by 40 human volunteers using formulation as lipo-lotion supplemented with 0.5% and 1% of coriander oil. CEO-supplemented Lipo-lotion effectively lowered the UV-induced erythema but was less effective than hydrocortisone. Yet, coriander oil showed a minor anti-inflammatory effect with good skin tolerance at both concentrations [53].

It was comprehended in our study that the treatment of JCEO was safe for RAW264.7 murine macrophages’ viability below concentrations of 0.6mg/mL. At a concentration of 0.3mg/mL, it was perceived as nontoxic and might be considered as metabolic enhancer according to MTT results- in terms of number of cells and/or intracellular activity. This was correlated to the scratch-gap closure overnight using the scratch wound assay. For the viability results, usually MTT dye is reduced by active mitochondria in living cells [59], however, some data suggest the reduction process occurs in the intracellular vesicles (such as endosomes or lysosomes), which later contribute to the needle-like aggregates of MTT formazan at the cell surface [59,60]. In our study results pertaining the high surge in the MTT assay at 0.3mg/mL JCEO treatment suggesting higher intracellular metabolism activity [59,60].

To consider coriander essential oil safety, there have been inconsistent reports on the mutagenicity of coriander components. As reported in one study, coriander ethanolic seeds extract failed to show any mutagenicity in rat embryo fibroblasts [61]. The principal component of coriander essential oil, linalool, was found to be antimutagenic at 25 mg/mL, and it did not encourage any chromosomal alterations in the Chinese hamster fibroblast assay [62]. Yet, the mutagenicity of coriander has been reported with an extract of coriander fruit showing mutagenic activity in the Ames assay involving some *Salmonella typhimurium* strains (TA98 and TA100) [61,63,64]. On the other hand, for the toxicity associated with the topical application of the coriander oil and its components, there seems to be adequate suggestions that the essential oil does not irritate the skin, not even after applying it for 48-hrs in a closed patch test with human volunteers [61,65,66].

To conclude, it is important to consider the presence of diverse range of compounds in the CEO which act in synergism to produce such bioactivities [67]. In that regard, examining the mechanisms of action of the JCEO constituents, may help towards the understanding of the interconnection between the composition and the related biological activity.

## Conclusion

The seed-extracted JCEO demonstrated a significant dose-response in free radical scavenging, reaching up to 98.7% at 100 mg/mL. Additionally, JCEO exhibited strong anti-inflammatory effects, with 0.3 mg/mL inducing the highest cytokine release. It also significantly inhibited LPS-induced NO and TNF-α production at 0.3 mg/mL and 0.16 mg/mL, respectively. Notably, 0.3 mg/mL of JCEO was the least toxic to RAW264.7 murine macrophages and may act as a potent anti-inflammatory agent. Collectively, these findings highlight JCEO’s potential as a therapeutic compound with strong antioxidant and anti-inflammatory properties.

## Supporting information

S1 File (DOC)
