# Supplementary material for: The effect of Jordanian essential oil from coriander seeds on antioxidant, anti-inflammatory, and immunostimulatory activities using RAW 246.7 murine macrophages
Source: PLoS One. 2024 Aug 6;19(8):e0297250. doi: 10.1371/journal.pone.0297250 (PMC11302854; doi:10.1371/journal.pone.0297250)
Supplement: S1 File — (DOC) [file pone.0297250.s001.doc]

# Acetone blank

GC-MS analysis

TIC plot

# JA-ESO

GC-MS analysis

TIC plot

A

B

C

D

E

F

G

GC Trace Peak A

# JA-ESO

GC-MS analysis

GC Trace Peak B

# JA-ESO

GC-MS analysis

GC Trace Peak C

# JA-ESO

GC-MS analysis

GC Trace Peak D

# JA-ESO

GC-MS analysis

GC Trace Peak E

# JA-ESO

GC-MS analysis

GC Trace Peak F

# JA-ESO

GC-MS analysis

GC Trace Peak G

# JA-ESO

GC-MS analysis
